# Effect of Magnolol on the Function of Osteoblastic MC3T3-E1 Cells

**DOI:** 10.1155/2012/829650

**Published:** 2012-02-09

**Authors:** Eun Jung Kwak, Young Soon Lee, Eun Mi Choi

**Affiliations:** ^1^Department of Food Science Technology, Yeungnam University, Gyeongsan 712-749, Republic of Korea; ^2^Department of Food & Nutrition, Kyung Hee University, 1 Hoegi-dong, Dongdaemun-gu, Seoul 130-701, Republic of Korea

## Abstract

*Objectives.* In the present study, the ability of magnolol, a hydroxylated biphenyl compound isolated from *Magnolia officinalis*, to stimulate osteoblast function and inhibit the release of bone-resorbing mediators was investigated in osteoblastic MC3T3-E1 cells. *Methods.* Osteoblast function was measured by cell growth, alkaline phosphatase activity, collagen synthesis, and mineralization. Glutathione content was also measured in the cells. Bone-resorbing cytokines, receptor activator of nuclear factor-**κ**B ligand (RANKL), TNF-**α**, and IL-6 were measured with an enzyme immunoassay system. *Results.* Magnolol caused a significant elevation of cell growth, alkaline phosphatase activity, collagen synthesis, mineralization, and glutathione content in the cells (*P* < 0.05). Skeletal turnover is orchestrated by a complex network of regulatory factors. Among cytokines, RANKL, TNF-**α**, and IL-6 were found to be key osteoclastogenetic molecules produced by osteoblasts. Magnolol significantly (*P* < 0.05) decreased the production of osteoclast differentiation inducing factors such as RANKL, TNF-**α**, and IL-6 in the presence of antimycin A, which inhibits mitochondrial electron transport and has been used as an ROS generator. *Conclusion.* Magnolol might be a candidate as an agent for the prevention of bone disorders such as osteoporosis.

## 1. Introduction

Bone tissue is continuously replaced through bone formation by osteoblasts and bone resorption by osteoclasts [1]. Both systemic factors and factors produced in the bone microenvironment are involved in the complex network regulating bone metabolism. Some of the locally produced factors involved in prevention of imbalances between bone formation and bone resorption also play prominent roles in diseases characterized by inflammation and increased bone resorption activity. TNF-*α* and IL-6 are proinflammatory cytokines produced by osteoblasts/stromal cells and well-known stimulator of bone resorption [2]. As a result, the number of osteoclasts is increased, leading to bone resorption, and osteoblast activity is repressed, leading to decreased mineralization. Receptor activator of nuclear factor-*κ*B ligand (RANKL) is a member of the tumor necrosis factor superfamily that is expressed in osteoblasts. RANKL, secreted mainly by osteoblastic stromal cells, is necessary for osteoclast formation from its committed precursors, which bear its receptor RANK. Activation of RANK leads to activation of downstream signaling pathways including NF-*κ*B, p38 kinase, and c-Jun N-terminal kinase (JNK) [3]. The RANKL: RANK signaling pathway could be a major target of antiresorptive agents.

*Magnolia officinalis* (Magnoliaceae) has long been used for the treatment of fever, headache, anxiety, diarrhea, asthma, and stroke and possesses potent anti-inflammatory effects [4]. It has been reported that magnolol, a compound purified from *Magnolia officinalis*, relaxes rat vascular smooth muscle [5], scavenges hydroxyl radicals [6], inhibits neutrophil aggregation and superoxide anion generation [7, 8], suppresses the expression of vascular cell adhesion molecule-1 in endothelial cells [9], and inhibits nitric oxide (NO) production in lipopolysaccharide- (LPS-) activated macrophages [10]. Previous studies showed that magnolol could attenuate peroxidative damage and improve the survival of rats after surgically induced sepsis [11] or sepsis-induced haemorrhagic shock [12]. Since magnolol has been reported to have antioxidant effect [13], it seems to have an effect on age-related osteoporosis. In the previous study, it was demonstrated that apocynin, a naturally occurring methoxy-substituted catechol, caused a significant elevation of osteoblast differentiation and decreased the production of ROS and osteoclast differentiation inducing factors in MC3T3-E1 cells [14].

The preosteoblastic MC3T3-E1 cell undergoes a temporal pattern of osteoblast development similar to *in vivo *bone formation. Thus, this cell is a well-accepted model of osteogenesis *in vitro *[15]. During the proliferative phase, this cell undergoes DNA synthesis and cell division, resulting in a rapid increase in cell number until confluence. At this juncture, proliferation is arrested, and there is an increase in the sequential expression of mature osteoblastic characteristics including alkaline phosphatase (ALP) production, conversion of procollagen to collagen, and the deposition of extracellular matrix on the substrate, which is subsequently mineralized [16]. In this study, to clarify the role of magnolol in bone formation and growth, the effects of magnolol on the proliferation and differentiation of osteoblastic cell lines were investigated using MC3T3-E1 *in vitro.* We also investigated whether magnolol inhibits the induction of bone resorbing mediators by antimycin A, which inhibits mitochondrial electron transport and has been used as an ROS generator.

## 2. Experimental Methods

### 2.1. Materials

Magnolol isolated from *Magnolia officinalis* was purchased from ChromaDex Inc. (Irvine, CA, USA) and antimycin A was purchased from Sigma Chemical (St. Louis, MO, USA). These were dissolved in dimethylsulfoxide (DMSO) and then diluted with the medium (final DMSO concentration ≤ 0.05% (v/v)). *α*-Modified minimal essential medium (*α*-MEM) and fetal bovine serum (FBS) were purchased from Gibco BRL (Grand Island, NY, USA). Other reagents were of the highest commercial grade available and purchased from Sigma Chemical (St. Louis, MO, USA).

### 2.2. Cell Culture

MC3T3-E1 cells (RCB1126, an osteoblast-like cell line from C57BL/6 mouse calvaria) were obtained from the RIKEN Cell Bank (Tsukuba, Japan). MC3T3-E1 cells were cultured at 37°C in 5% CO_2_ atmosphere in *α*-modified minimal essential medium (*α*-MEM; GIBCO). Unless otherwise specified, the medium contained 10% heat-inactivated fetal bovine serum (FBS), 100 U/mL penicillin, and 100 mg/mL streptomycin.

### 2.3. Cell Growth

The cells were suspended in medium and plated at a density of 10^4^ cells/well into a 24-well culture dish (Costar, Cambridge, MA, USA). After 48 h, the medium was replaced with serum-free media containing 0.3% bovine serum albumin supplemented with magnolol. After 2 days of culture, cell growth was measured by MTT assay. This assay is based on the ability of viable cells to convert soluble 3-(4,5-dimethyl-thiazol-2yl)-2,5-diphenyl tetrazolium bromide (MTT) into an insoluble dark blue formazan reaction product. MTT 20 *μ*l in 7.2 mM phosphate buffer solution, pH 6.5 (5 mg/mL), was added to each well, and the plates were incubated for an additional 2 h. After the removal of solutions in the well, dimethyl sulfoxide was added to dissolve formazan products, and the plates were shaken for 5 min. The absorbance of each well was recorded on a microplate spectrophotometer at 570 nm.

### 2.4. Collagen Content

The cells were treated, at confluence, with culture medium containing 10 mM *β*-glycerophosphate and 50 *μ*g/mL ascorbic acid (differentiation medium) to initiate differentiation. After 6 days, the cells were incubated with magnolol for 48 h. Collagen content was quantified by Sirius Red-based colorimetric assay. Cultured osteoblasts were washed with PBS, followed by fixation with Bouin's fluid for 1 h. After fixation, the fixation fluid was removed and the culture dishes were washed by immersion in running tap water for 15 min. The culture dishes were air dried and stained by Sirius Red dye reagent for 1 h under mild shaking on a shaker. Thereafter, the solution was removed, and the cultures were washed with 0.01 N HCl to remove nonbound dye. The stained material was dissolved in 0.1 N NaOH, and absorbance was measured at 550 nm.

### 2.5. Alkaline Phosphatase Activity

The cells were treated, at confluence, with differentiation medium to initiate differentiation. After 6 days, the cells were incubated with magnolol for 48 h. The cells were lysed with 0.2% Triton X-100, with the lysate centrifuged at 14,000 ×g for 5 min. The clear supernatant was used to measure the ALP activity, which was determined using an ALP activity assay kit (Asan Co. Korea). Protein concentrations were determined using the BioRad protein assay reagent.

### 2.6. Calcium Deposition Assay

The cells were treated, at confluence, with differentiation medium. After 14 days, the cells were cultured with medium containing magnolol for 2 days. On harvesting, the cells were fixed with 70% ethanol for 1 h and then stained with 40 mM Alizarin Red S for 10 min with gentle shaking. To quantify the bound dye, the stain was solubilized with 10% cetylpyridinium chloride by shaking for 15 min. The absorbance of the solubilized stain was measured at 561 nm.

### 2.7. Intracellular Glutathione Measurement

The cells were treated, at confluence, with differentiation medium to initiate differentiation. After 6 days, the cells were incubated with magnolol for 48 h. Cells were lysed by homogenization in 1-2 mL of cold buffer containing 50 mM MES or phosphate (pH 6-7) and 1 mM EDTA. After centrifugation at 10,000 g for 15 min at 4°C, supernatant was used for assay. Glutathione was measured by use of the Glutathione Assay Kit (BioAssay Systems, Hayward, CA, USA) according to manufacturer's instructions. Determination of glutathione is based on the reaction of 5, 5′-dithiobis-2-nitrobenzoic acid (DTNB) with glutathione which yield a yellow-colored chromophore, 5-thionitrobenzoic acid (TNB) with a maximum absorbance at 412 nm. Concentrations of glutathione were determined from a freshly prepared standard curve of glutathione.

### 2.8. Measurement of RANKL, TNF-*α*, and IL-6

The cells were treated, at confluence, with differentiation medium to initiate differentiation. After 6 days, the cells were pre-incubated with magnolol for 1 h before treatment with antimycin A for 48 h. RANKL, TNF-*α*, and IL-6 contents in the medium were measured with an enzyme immunoassay system (R&D system Inc., Minneapolis, MN, USA) according to the manufacturer's recommendation.

### 2.9. Statistical Analysis

All experiments were carried out in triplicate, and all results are expressed as mean ± SEM of at least 3 independent experiments. Statistical significance was determined by analysis of variance and subsequently applying Dunnett's *t*-test (*P* < 0.05).

## 3. Results

### 3.1. Effect of Magnolol on the Growth and Differentiation of MC3T3-E1 Cells

**Figure 1:**
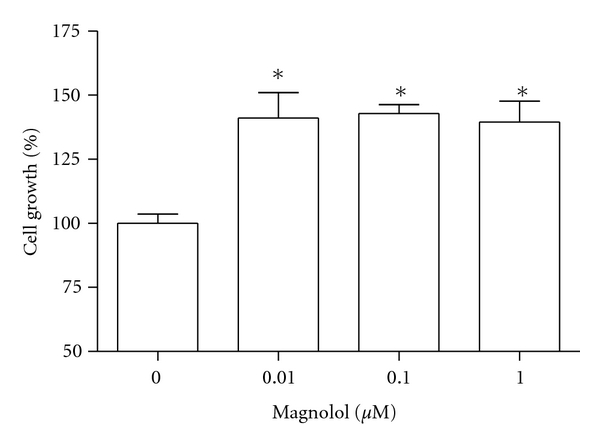
Effect of magnolol on the growth of MC3T3-E1 cells. Data were expressed as a percentage of control. *P < 0.05 compared with control.

**Figure 2:**
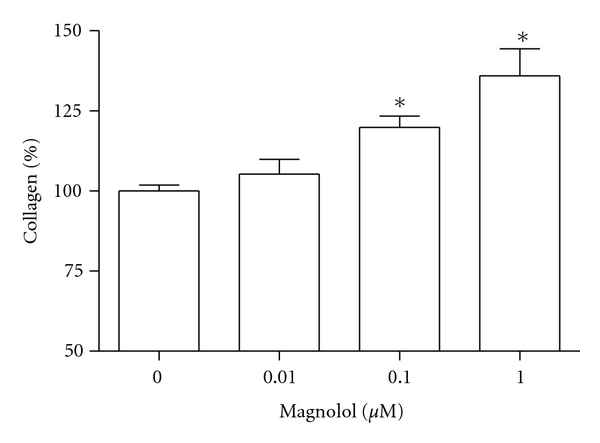
Effect of magnolol on the collagen synthesis of MC3T3-E1 cells. Data were expressed as a percentage of control. The control value for collagen content was 23.45 ± 0.659 *μ*g/10^6^ cells. *P < 0.05 compared with control.

**Figure 3:**
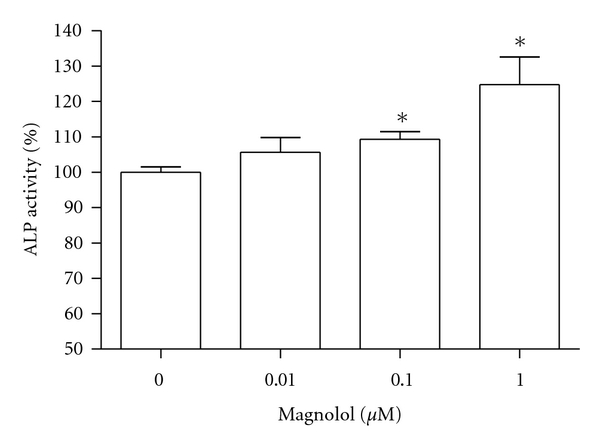
Effect of magnolol on the alkaline phosphatase activity of MC3T3-E1 cells. Data were expressed as a percentage of control. The control value for ALP activity was 0.871 ± 0.016 Unit/mg. *P < 0.05 compared with control.

**Figure 4:**
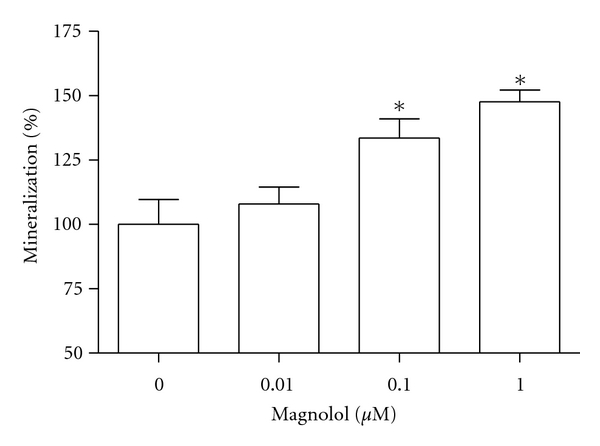
Effect of magnolol on the mineralization of osteoblastic MC3T3-E1 cells. Data were expressed as a percentage of control. The control value for mineralization was 0.504 ± 0.006 OD/10^6^ cells. *P < 0.05 compared with control.

MC3T3-E1 cells were incubated with magnolol and cell growth was measured. Cell populations cultured in basal or magnolol-treated media appeared as Figure 1. MC3T3-E1 cell growth was promoted by stimulation with magnolol (0.1~1 *μ*M) significantly compared with control cells. The effect of magnolol on collagen synthesis in osteoblastic MC3T3-E1 cells is shown in Figure 2. The collagen synthesis of MC3T3-E1 cells was significantly increased by the addition of 0.1~1 *μ*M magnolol. ALP activity was measured to study the effect of magnolol on the osteoblastic differentiation in MC3T3-E1 cells. The cultured cells in the presence of magnolol (0.1 and 1 *μ*M) caused a significant increase in the ALP activity of osteoblastic cells (Figure 3). The MC3T3-E1 cells were treated with various concentrations of magnolol, and the mineralization of osteoblasts was measured. The increase of mineralization was significant at magnolol concentrations of 0.1 and 1 *μ*M in MC3T3-E1 cell culture (Figure 4).

### 3.2. Effect of Magnolol on the Intracellular Glutathione Content of MC3T3-E1 Cells

**Figure 5:**
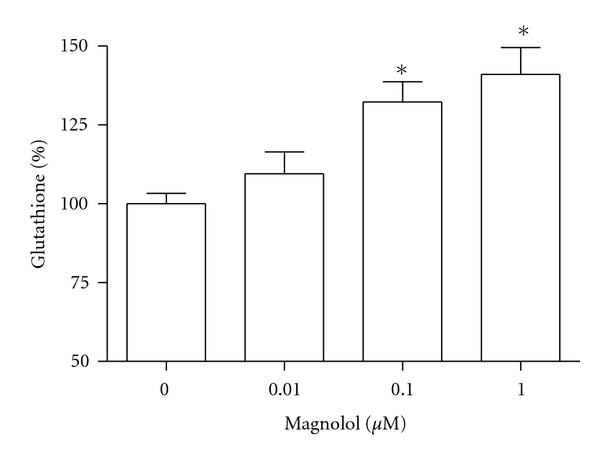
Effect of magnolol on the glutathione content of MC3T3-E1 cells. Data were expressed as a percentage of control. The control value for collagen content was 12.8 ± 0.305 nmole/mg. *P < 0.05 compared with control.

Glutathione content reflects the amount of substrate available to act as antioxidant. The level of glutathione after magnolol exposure for 48 h was significantly increased at the concentration of 0.1 and 1 *μ*M as compared with control, indicating that magnolol-induced enhancements of osteoblast function are associated with an increase in glutathione (Figure 5).

### 3.3. Effect of Magnolol on RANKL Production of MC3T3-E1 Cells in the Presence of Antimycin A

**Figure 6:**
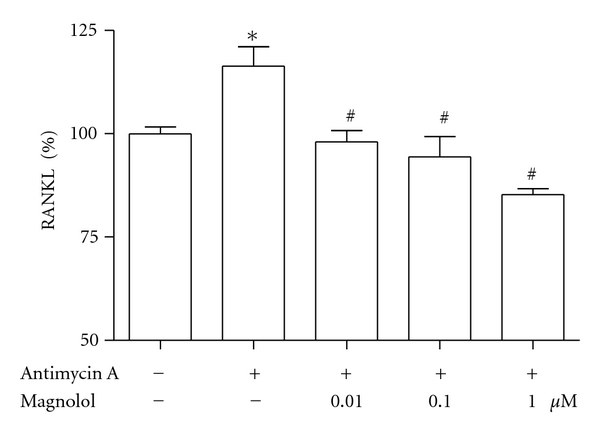
Effect of magnolol on antimycin A-induced RANKL production of MC3T3-E1 cells. Effect of magnolol on the production of RANKL in the presence of antimycin A. Osteoblasts were pre-incubated with magnolol before treatment with 70 *μ*M antimycin A for 48 h. Data were expressed as a percentage of control. The control value for RANKL was 3.378 ± 0.074 ng/mg. *P < 0.05, control versus antimycin A; ^#^ *P* < 0.05, antimycin A versus magnolol.

In order to further determine the regulator of osteoclast differentiation in osteoblasts, we examined the production of RANKL in the osteoblastic MC3T3-E1 cells. When 70 *μ*M antimycin A was added to cells, the production of RANKL increased significantly (Figure 6). However, antimycin A-induced RANKL production was significantly inhibited by treatment of magnolol (0.01~1 *μ*M).

### 3.4. Effect of Magnolol on Antimycin A-Induced TNF-*α* and IL-6 Production in MC3T3-E1 Cells

**Figure 7:**
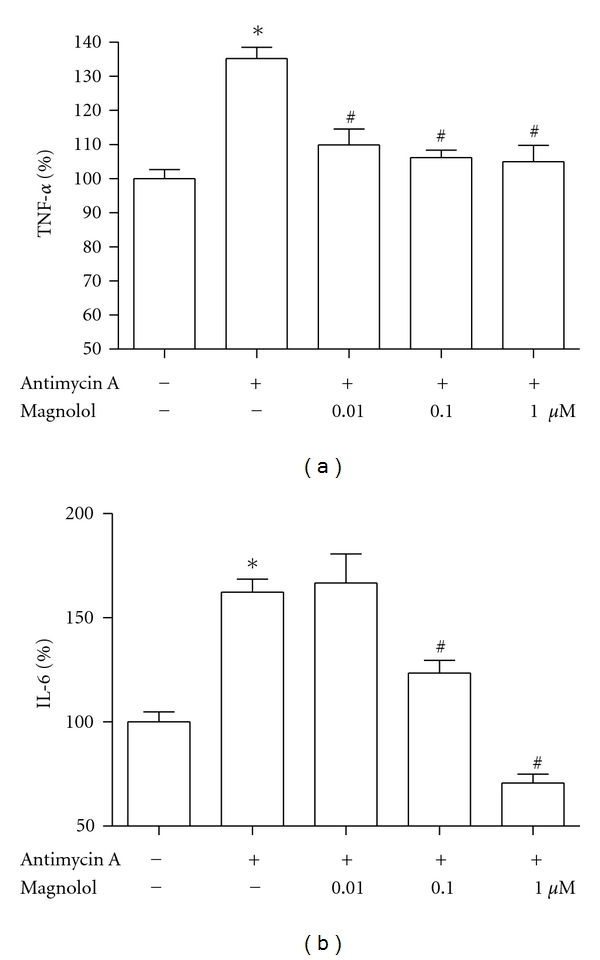
Effect of magnolol on antimycin A-induced cytokines production of MC3T3-E1 cells. Osteoblasts were preincubated with magnolol before treatment with 70 *μ*M antimycin A for 48 h. Data were expressed as a percentage of control. The control values for TNF-*α* (a) and IL-6 (b) were 0.228 ± 0.006 ng/mg and 0.655 ± 0.033 ng/mg, respectively. *P < 0.05, control versus antimycin A; ^#^ *P* < 0.05, antimycin A versus magnolol.

TNF-*α* and IL-6 have been demonstrated to increase osteoclastic activity. Thus, we also investigated whether magnolol modulates antimycin A-induced production of TNF-*α* and IL-6 (Figure 7). When 70 *μ*M antimycin A was added to cells, production of TNF-*α* and IL-6 increased significantly. However, antimycin A-induced TNF-*α* and IL-6 productions were significantly inhibited by treatment of magnolol at 0.01~1 *μ*M and 0.1~1 *μ*M, respectively.

## 4. Discussion

The osteoblast phenotypes are acquired in two stages. In the first stage, the matrix matures and specific proteins associated with the bone cell phenotype, such as collagen and ALP, are detected. In the second stage, matrix becomes mineralized by calcium deposition. As a result, layers of spongy bone are formed around the original cartilage. Later in development, spaces among the spongy bone are filled with bone matrix and become compact bone [17]. Hence, collagen content and ALP activity, an early differentiation marker, and cellular calcium content, a late marker of differentiation, were examined to investigate the effects of magnolol on the differentiation of osteoblast cells. In this study, it was found that magnolol markedly increased osteoblast growth and differentiation in osteoblastic MC3T3-E1 cells. The findings of the present study supported our hypothesis that magnolol increases the osteogenic effect in osteoblastic MC3T3-E1 cells and these stimulated osteogenic effects are mediated by increasing osteoblast proliferation and differentiation. Findings from this study show that magnolol increased osteoblast proliferation during the early osteoblast proliferation phase. During the early proliferation period, osteoblasts synthesize and secrete cell-growth proteins such as TGF-*β* or IGF [18] and collagen, which is the most abundant protein in extracellular matrix accounting for matrix maturation [19]. Therefore, we suggest that increased osteoblast proliferation by magnolol indicates an anabolic effect on the bone matrix formation by stimulating osteoblast cell growth rate and by increasing collagenous protein synthesis which is a critical factor for matrix maturation.

Oxidative stress [20] and changes in the bone microenvironment composition with aging [21] may play an important role in the pathogenesis of postmenopausal osteoporosis by impairing osteoprogenitor cell recruitment and differentiation. Jiang et al. [22] studied *in vitro* the characteristics of alveolar osteoblasts from elderly women and revealed differences in proliferative capacity and bone formation functions, which dropped with aging. In this context, an important correlation has been shown between oxidative stress and postmenopausal bone loss occurrence during aging. Maggio et al. [23] reported that antioxidant defenses are markedly decreased in plasma of osteoporotic women. In agreement, Altindag et al. [24] associated this imbalance between oxidant and antioxidant status in postmenopausal osteoporosis with increase of osteoclastic activity and decreased osteoblastic activity. Reduced glutathione is considered one of the most important intracellular reducer agents. This tripeptide is involved in the protection against cytotoxicity electrophilic agents and metabolites, and it is also involved with the regulation of the effects of oxidative stress on the cells, maintaining, in this way, the intracellular redox balance [25]. Glutathione is considered to be responsible for reactivation of some proteins after suffering oxidative stress [26]. In the presence of glutathione, the sulfenic derivative can be converted in a more stable product, preventing further oxidation [27]. In the present study, the level of glutathione after magnolol exposure was significantly increased. Our result shows that the enhancement of antioxidants by magnolol may be related with the increased function of osteoblasts.

Apart from having an effect on bone formation, osteoblasts are also coupled with osteoclasts through the release of various cytokines, including RANKL [28]. Most proosteoclastogenic cytokines act primarily through osteoblasts to alter levels of RANKL. RANKL is a protein expressed on the osteoblast cell membrane that binds to its cognate receptor RANK, which is present on the osteoclast progenitor membrane. The binding of RANKL to RANK activates nuclear factor-*κ*B and c-jun N-terminal protein kinase, which is associated with osteoclastic differentiation and activation [29]. Oxidative stress plays an important role in the destruction of bone cells during the development of osteoporosis. Impaired mitochondrial function can lead to increased ROS generation and may increase oxidative stress if the antioxidant defense mechanisms of the cells are overwhelmed [30]. Increased oxidative stress caused by mitochondrial dysfunction is considered the causal link between elevated ROS and the major biochemical pathways postulated to be involved in the pathogenesis of senile disorders [31]. Antimycin A is an inhibitor of mitochondrial electron transport via its binding to complex III [32]. In this study, magnolol inhibited the production of RANKL induced by antimycin A in osteoblastic cells. Dong et al. [33] reported that 3,3′-diindolmethane, one of the natural compounds formed during the autolysis of glucobrassicin, effectively inhibited the expression of RANKL in osteoblastic cells, leading to the blockade of osteoclastogenesis and consequently an alleviation of experimental arthritis. Because RANKL is also known to induce actin ring formation in mature osteoclasts, magnolol-induced inhibition of RANKL release in osteoblastic cells could result in disruption of actin tings in osteoclasts.

Several family members are produced by bone cells and have been implicated to be involved in regulation of bone metabolism. In the present paper, we show that TNF-*α* and IL-6 releases induced by antimycin A were decreased by magnolol. The inhibitory effect on TNF-*α* and IL-6 production by magnolol may contribute to the bone antiresorbing effect of magnolol and possibly also play a role in the reduction of bone loss seen in the vicinity of inflammatory processes such as rheumatoid arthritis and periodontal disease. Although the precise and detailed mechanism of magnolol inhibition remains to be scrutinized, we propose that magnolol might be useful as a potential therapeutic medication for attenuating osteoclast formation and function in the prevention and treatment of bone diseases such as osteoporosis. That TNF-*α* can inhibit osteoblast differentiation, and bone formation is well known. Using two models of osteoblast differentiation, Gilbert et al. [34] found that TNF-*α* inhibited cell differentiation by downregulating the transcription of Runx2, which regulates the expression of bone matrix proteins. RUNX2 protein stability has also been shown to be destabilized by TNF-*α* [35]. Hughes and Howells [36] treated osteoblasts derived from fetal rat calvaria with recombinant human IL-6 and showed that bone nodule formation was inhibited. Fang and Hahn [37] examined the effects of IL-6 in UMR-106–01 cells, a rat osteoblastic osteosarcoma line, and observed that it suppressed collagen synthesis. IL-6 may have a role in the osteopenia associated with inflammation *in vivo*. Patients with inflammatory diseases in whom IL-6 levels are highest may be at increased risk for osteopenia.

In summary, magnolol increased the proliferation of osteoblasts and stimulated ALP activity and bone matrix proteins, such as collagen, and these increases trigger osteoblastic differentiation (e.g., mineralized nodule formation). Moreover, our data indicate that magnolol suppresses the production of the bone-resorbing factors in the presence of antimycin A, a mitochondrial inhibitor that increases the generation of ROS. Thus, magnolol may be a good candidate for the protection of osteoblast dysfunction. These results may aid in the development of a therapeutic approach of magnolol in the prevention of osteoporosis.
